# Do goldfish like to be informed?

**DOI:** 10.1098/rspb.2024.2842

**Published:** 2025-05-21

**Authors:** Victor Ajuwon, Tiago Monteiro, Mark E. Walton, Alex Kacelnik

**Affiliations:** ^1^ Department of Biology, University of Oxford, Oxford OX1 3RB, UK; ^2^ Department of Psychology, University of Cambridge, Cambridge CB2 3EB, UK; ^3^ William James Center for Research, University of Aveiro, Aveiro 3810-193, Portugal; ^4^ Department of Education and Psychology, University of Aveiro, Aveiro 3810-193, Portugal; ^5^ Domestication Lab, Department of Interdisciplinary Life Sciences, University of Veterinary Medicine Vienna, Vienna 1210, Austria; ^6^ Department of Experimental Psychology, University of Oxford, Oxford OX2 6GG, UK

**Keywords:** information-seeking, non-instrumental information, observing response, paradoxical choice, suboptimal choice, goldfish

## Abstract

Like humans, several mammalian and avian species prefer foretold over unsignalled future events, even if the information is costly and confers no direct benefit. It is unclear whether this is an epiphenomenon of basic associative learning mechanisms, or whether these preferences reflect a derived form of information-seeking that is reminiscent of human curiosity. We investigate whether a fish that shares basic reinforcement learning mechanisms with birds and mammals also shows such a preference, with the aim of elucidating whether widely shared conditioning processes are sufficient to explain paradoxical preferences resulting in unusable information. Goldfish (*Carassius auratus*) chose between two alternatives, both resulting in a 5 s delay and 50% reward chance. The ‘informative’ option immediately produced a stimulus correlated with the trial’s forthcoming outcome (reward/no reward). Choosing the ‘non-informative’ option instead triggered an uncorrelated stimulus. Goldfish discriminated between the different contingencies but did not develop a preference for the informative option, suggesting that in goldfish associative learning mechanisms are not sufficient to generate preferences between alternatives differing only in outcome predictability. These results challenge the notion that informative preferences are a by-product of ubiquitous associative processes, and are consistent with the possibility that derived information-seeking mechanisms have evolved in some vertebrate species.

## 1. Introduction

Prospective information enables organisms to exploit opportunities, increasing the acquisition of essential resources such as food, while reducing undesirable risks. The fact that information is valuable inasmuch as it can lead to adaptive behavioural change is part of normative accounts of learning (for a review, see [1]) and decision-making, in a range of fields including microeconomics [2], neuroeconomics [3] and behavioural ecology [4,5]. However, experimental results show that informative stimuli can reinforce the behaviour of animals in the laboratory, regardless of whether this correlates with substantial gains in primary reinforcement such as food or water rewards [6–9]. Bridging the gap between the normative rationale of reward-maximization prevalent in decision-making theories, and empirical results demonstrating the reinforcing effect of informative stimuli has widespread interest for behavioural biology and comparative cognition.

In an experimental paradigm variously referred to as ‘paradoxical choice’, ‘suboptimal choice’ and ‘non-instrumental information seeking’, humans [10–14] and a range of mammalian [6,15–19] and avian [20–24] species prefer choosing an alternative that provides informative signals about forthcoming outcomes (i.e. food/no food), even though in the experimental protocols the information cannot be used to modify outcomes and may come at considerable cost (see also ‘observing responses’ [8]). Subjects are typically presented with two options that deliver food or liquid rewards probabilistically, a fixed delay after the choosing response. In the informative option (*Info*), either of two outcome-predictive stimuli are immediately presented after each choice, one paired with a forthcoming reward and the other with no reward. In the uninformative option (*NoInfo*), outcomes are not differentially signalled, and food is either delivered or not once the delay has lapsed, with subjects uncertain during the delay. The probabilities of food/no food vary depending on the implementation. Critically, subjects cannot exploit the information in the *Info* alternative because it is supplied during a waiting delay after they have chosen, hence the information’s label as ‘non-instrumental’ by some authors. In one striking example, starlings (*Sturnus vulgaris*) significantly preferred an option that gave food on 5% of trials over an alternative that gave food on 50% of trials, when the only additional difference was that in the leaner option the outcome was signalled during the 10 s of waiting between choice and outcome [24].

Recent experimental observations and theoretical models support the view that preference in paradoxical choice arises because individuals ascribe value to stimuli because they reduce uncertainty, independent of associated instrumental utility [25–28]. In particular, some authors argue that the observed preference for the informative option reflects intrinsically motivated information-seeking mechanisms [10,12,26,29]. Neuroscientific studies on macaques (e.g. [30,31]) have helped to advance the notion that preferences in such tasks reflect behaviour that is an analogue (or homologue) to human curiosity, reflecting psychological mechanisms aimed at resolving uncertainty [10,14,32] or filling ‘information gaps’ [33]. This view is supported by evidence in both humans [12,34,35] and monkeys [15,16,34] showing that the same midbrain dopamine neurons can encode both information gain and primary rewards such as food. According to this view, while non-instrumental information-seeking does not result in immediate, tangible benefits, in the laboratory choice tasks, this drive enables individuals to acquire information about the environment that may be useful during problem-solving in the future [36]. However, there are alternative interpretations.

Studies on pigeons have helped to characterize a range of experimental factors that influence preference in paradoxical choice tasks [8,21,37,38], and generally advance an alternative account of observed preferences that do not posit a sensitivity to uncertainty reduction *per se*. According to this view, Pavlovian stimuli paired with positive outcomes become appetitive, conditioned reinforcers, while stimuli paired with negative outcomes (such as omission of food delivery) are weakly inhibitory or ignored altogether [8,23,37,39]. If signals for positive outcomes cause a greater increase in the probability of the preceding choice than the decrease caused by signals for negative outcomes, then the net result can differentially favour the ‘informative’ option, just by the asymmetric effect of conditioned reinforcement. In other words, individuals select *Info* to generate appetitive ‘good news’ cues, rather than to reduce uncertainty. This is an appealing, parsimonious interpretation that does not rely on uncertainty-related cognition, but applies less convincingly to cases where individuals sacrifice significant quantities of primary reward, since this would require the reinforcing effect of secondary reinforcers to significantly exceed that of traditional primary rewards. Similarly, a study on humans has suggested that ‘good news’ stimuli derive value by allowing subjects to hedonically savour forthcoming outcomes while they wait for them [13]. This requires that the ‘savouring’ effect in the presence of signals for positive outcomes is stronger than the aversive ‘dreading’ effect of a foretold failure to receive reward. Proponents of these views have also suggested that the underlying mechanisms reflect unavoidable cognitive constraints that are also implicated in pathological human gambling [13,40–43], and indicate ‘suboptimal’ behaviour, but this is debated. Valence-dependent asymmetries in learning are also consistent with a theoretical model inspired by optimal foraging theory, suggesting that the observed preferences are adaptive, reflecting mechanisms that would maximize reward rate in natural foraging scenarios when prospective information about rewards is usable [24].

Though preferences for alternatives providing prospective information are now well established in some animal species, it remains unclear whether the preferences are a by-product of widely shared features of reinforcement learning—secondary reinforcement from appetitive cues—or whether this reflects an adaptive drive to reduce uncertainty [7,44]. So far, studies have been restricted to a relatively small set of mammal and bird species, primarily macaques, rats, starlings and pigeons. In contrast, basic associative processes are known to be shared across vertebrates. Therefore, showing that the paradoxical observations are replicable in a wider range of species endowed with ordinary reinforcement learning capabilities would favour the view that conditioned reinforcement is sufficient to generate apparent information-seeking behaviour, while the need to invoke a direct appetitive influence of abstract representations such as uncertainty to explain paradoxical choice would be weakened. On the other hand, an absence of preference for the informative alternative in species that nonetheless possess basic reinforcement learning capabilities would suggest that observed preferences reflect derived information-seeking mechanisms unique to particular species and suggest that conditioned reinforcement is not sufficient.

Here, we address this by implementing, for the first time, the paradoxical choice protocol in a teleost fish, goldfish (*Carassius auratus*)—a species that is phylogenetically distant to those that have so far been tested. Goldfish are a suitable starting point to explore the widespread prevalence of paradoxical choice because, though relatively under-utilized, they are an established model in cognitive research [45,46], including in associative learning tasks [47–49], and their behaviour can be reinforced by conditioned stimuli [50], making them a good candidate to examine the subjective value of informative stimuli. Goldfish also diverge dramatically in brain architecture from the mammalian and avian species so far tested [51], and thereby provide an opportunity to test the conjecture that derived neocortex-like structures are critical to information-seeking behaviour [31].

## 2. Methods

### (a) Subjects

Eight goldfish ranging in size between 7 and 10 cm (age and sex unknown) participated. They were obtained from a local commercial supplier (Goldfish Bowl, Oxford, UK) and housed in groups of two or three, in holding aquaria (60 cm × 35 cm × 31 cm; length × width × height) where they had access to a rock shelter, pebbles and artificial plants. Individuals participated in experiments five times a week on weekdays and were fed *Fancy Goldfish Sinking Pellets* during sessions. This diet was supplemented with spinach following experiments on the last day of the working week and bloodworms the day after (subjects were not directly fed on Sundays). Fish were kept under a 12:12 h light:dark cycle using fluorescent lights. Water was maintained at a minimum of 21℃ using an internal heater and independent thermometer (pH: 8.2; ammonia: 0 ppm; nitrite: 0 ppm; nitrate: max. 30 ppm). Partial water changes were conducted at the end of each week, and internal filters were cleaned every month. Each holding tank was aerated using an air pump.

For each daily session, fish were transported to their experimental tank and then back to their holding tank in a plastic jug. At the start of each day, approximately 20 l of water from the holding tanks was transferred to the experimental tanks in order to keep the environmental conditions as constant as possible. The experimental tanks were cleaned at the end of each week. The animals had different levels of previous experience in unrelated protocols (see [52]).

### (b) Apparatus and task control

Experiments were run using *GoFish*, an open-source experimental platform for closed-loop behavioural experiments on aquatic species [47,53]. The use of *GoFish* brings a similar level of automation and control to that found in previous paradoxical choice experiments with other species, aiding interspecies comparisons and reducing potential experimenter-induced bias.

**Figure 1.**
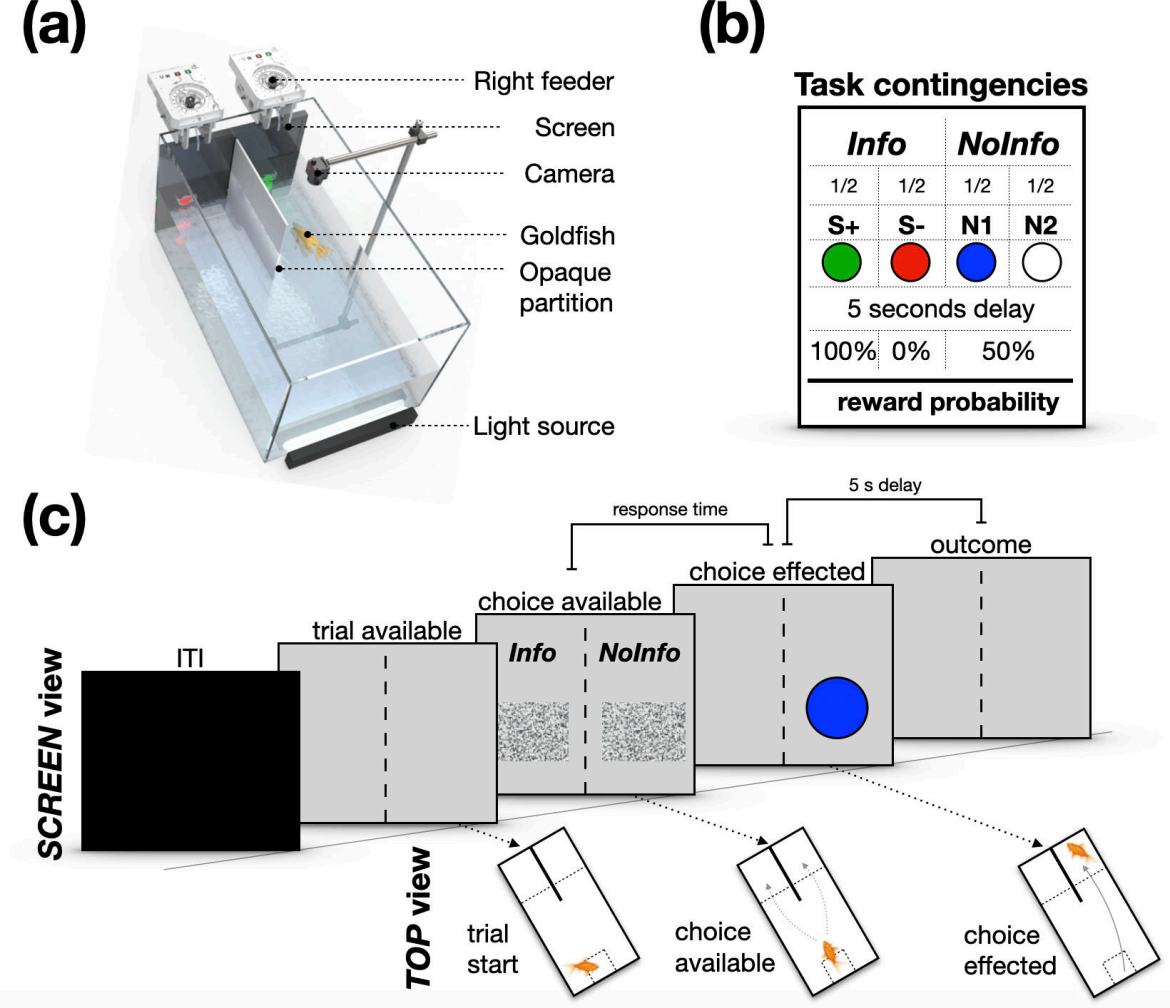
Exploring preference for advance information in goldfish. (a) The *GoFish* operant chamber includes a screen for stimulus presentation, two pellet dispensers placed on either side of an opaque acrylic divider in a y-maze configuration, an overhanging camera and an aquarium light. (b) In phase 1 of the experiment, each subject chose between two options: in *Info*, the forthcoming reward outcome was signalled immediately post-choice; in *NoInfo*, stimuli were not outcome predictive. (c) Illustrative example of a phase 1 trial showing a subject initiating the trial and choosing the non-informative option.

The experimental set-up constituted a rectangular tank (60 cm × 30 cm × 36 cm; length × width × height) with 15 cm depth of water; a 17″ LCD computer screen (1920 × 1080; 60 Hz) for stimulus presentation; two custom-made pellet dispensers placed on either side of an opaque acrylic divider in a y-maze configuration; an overhanging USB camera (1280 × 720 resolution) and an aquarium light (figure 1a). Each reward delivery consisted of one *Fancy Goldfish Sinking Pellet*. Experiments were run in parallel in two experimental tanks with four fish randomly assigned to each at the start of the experiment.

Task control was automated and implemented using a custom *Bonsai* workflow [54,55]. Task contingencies (i.e. stimulus presentation and reward delivery as a function of behaviour) were dependent on fish being within specified regions-of-interest (ROIs) in the experimental tank and were controlled using real-time video detection of fish movement. Within each tank, three ROIs were defined: a ‘start zone’, ‘left choice zone’ and ‘right choice zone’. These zones were not defined by physical boundary markings but were delineated on the video feed corresponding to fixed areas within the experimental tank. To detect fish presence in the ROIs, the following method was used. A video of the experimental tank was recorded at approximately 33 frames per second and converted to hue, saturation and value (HSV) colour space. An HSV range was applied to detect the fish. The pixels of the resulting binarized frames from each ROI were summed continuously. Fish entries into the zones were recorded when the summed ROI pixel value exceeded a pre-set threshold (ROI thresholds were adjusted to each subject prior to the onset of the experiment). Thus, subjects were able to control the progress of trials and make choices via their position in the tank. We also used colour thresholding to continuously track the centroid of each fish throughout experimental sessions (for further details see [47]).

### (c) Pre-training

Pre-training consisted of three phases: (i) experimental tank acclimatization, (ii) choice zone training, for subjects to learn that swimming into either the left or right choice zone (outside of the inter-trial interval, ITI) could be followed by food delivery, and (iii) start position training, for subjects to learn that a start position had to be entered before entry into choice zones was reinforced. Advancing through the phases depended on the individual subject’s performance. For further details, see ‘Pre-training’ in [47].

### (d) Paradoxical choice experiment

#### (i) Phase 1

We used a trial-based chain procedure. Every fish was presented with two daily sessions of 24 trials for 45 days. There were two kinds of trials: two-option choice trials and one-option forced trials. The proportion of each of these trials within a session changed as the experiment progressed (electronic supplementary material, table S1), but for the first 15 days, subjects were exposed only to forced trials. Subsequently, where a session was composed of both types of trial, they were randomly intermixed. Trials were separated by an ITI (drawn from a uniform distribution: min. = 20 s; max. = 40 s) during which the screen was black and behaviour had no consequences. The end of the ITI was signalled by a grey screen, and from this moment on, entering the start position would trigger the presentation of a visual white noise stimulus in the half screen facing one of the two choice zones (one-option forced trial) or both choice zones (two-option choice trial). Swimming into either choice zone with the white noise stimulus present led to the substitution of the white noise stimulus by a geometric shape visual stimulus (S+, S−, N1 or N2, see the ‘Stimuli’ section), which remained present for 5 s, until the end of the trial.

There were two options (figure 1b), which could either be presented alone (one-option forced trial) or simultaneously (two-option choice trial). Swimming into the choice zone of the informative option (*Info*) resulted with equal probability in either of the sequences ‘S+ ⇒ wait for 5 s ⇒ food’, or ‘S- ⇒ wait for 5 s ⇒ nothing’, thereby reliably informing the subject of the forthcoming outcome. Swimming into the non-informative (*NoInfo*) choice zone, on the other hand, resulted with equal probability in either of two sequences: ‘N1 ⇒ wait for 5 s ⇒ 50% either food or nothing’ or ‘N2 ⇒ wait for 5 s ⇒ 50% either food or nothing’; therefore, in *NoInfo* neither cue informed the subject of the forthcoming outcome, but reward probability was the same as when choosing *Info* (an example choice trial is shown in figure 1c). The sides of the *Info/NoInfo* options were consistent for each individual but counterbalanced across subjects.

#### (ii) Phase 2

After 30 days of being presented with choices between *Info* and *NoInfo*, subjects were presented with randomly intermingled choices between the following pairs of stimuli: S+ versus either N1 or N2 and S− versus either N1 or N2. The aim of this phase was to test whether the fish had learned the contingencies of the terminal links in the chain trials. There were two daily sessions of 24 trials per day, which lasted for 5 days. Trials were preceded by an ITI (drawn from a uniform distribution: min. = 20 s; max. = 40 s) where the screen was black and behaviour had no consequences. The end of the ITI was signalled by a grey screen, and from this moment on, entering the start position triggered the presentation of S+ or S− in one choice zone and N1 or N2 in the other. Swimming into a choice zone led to the reward outcome for the chosen stimulus according to the same contingencies as in *phase 1*, followed by the onset of the ITI.

### (e) Stimuli

In the pre-training phase, we used a monochromatic white noise rectangle (13.5 cm × 12 cm, Gaussian: mean = 0, variance = 10) to signal that reward delivery was contingent on the fish swimming to a specific location in the tank. In *phase 1* of the main experiment, the white noise cue was presented on either the right or left half of the screen (or both) to indicate whether the choice zones were primed to advance the protocol upon entering. During *phase 1* and *phase 2*, the outcome-predictive cues were four coloured circles presented on the screen, each paired with a reward probability (1, 0 or 0.5, for S+, S− and N1/N2, respectively, with colours counterbalanced across subjects). These were red, green, blue and white circles, 3.5 cm in diameter on a grey background, with centres positioned 5 cm from the bottom of the tank and 7 cm from each side wall. We chose colours that have been physiologically [56] and behaviourally [47,57] proven to be discernible by goldfish.

### (f) Data analysis

Data processing and analyses were carried out in MATLAB 2024b [58] (See electronic supplementary material, appendix 1, for details), while statistical analyses were conducted in RStudio (v. 1.2.5033 [59]). To normalize the residuals in statistical analyses, choice proportion data from *phase 1* and *phase 2* were arcsin square-root transformed, and response time data from *phase 1* were log transformed. A type I error rate of 0.05 was adopted for all statistical comparisons.

#### (i) Phase 1

Repeated measures ANOVAs were carried out on choice proportion data from the last 30 days of *phase 1* and response time data from the last 5 days of *phase 1*. We also carried out Bayesian analyses on choice proportion data to determine the strength of evidence obtained from the repeated measures ANOVA (see electronic supplementary material).

To examine whether subjects learned the contingencies of each outcome-predictive stimulus, we conducted a repeated measures ANOVA on ‘movement metric’ data from the last 5 days of *phase 1*. *Post hoc* pairwise comparisons using *t*-tests with a Bonferroni correction were also conducted. For statistical analyses, movement metric data were computed as follows. We quantified fish spatial occupancy in a 4 s time window preceding reward outcomes (excluding 0.5 s after choice and 0.5 s pre-outcome) using entropy (a measure of randomness) as a ‘movement metric’ to parametrize the spatial distribution of subjects. This allowed us to quantify subjects’ responses to the various outcome-predictive stimuli presented post-choice. The movement metric is given by:


$$movement\;metric=-\Sigma (P_{i}-log_{2}(P_{i}))$$

as implemented in the function entropy (*Entropy–File Exchange–MATLAB CentralFile Exchange* [60]), where *P* are matrices containing the normalized spatial histogram (3 cm^2^ bins) counts corresponding to subject occupancy for each stimulus type, S+, S−, N1 and N2, with N1 and N2 pooled together, since they have identical reward contingencies. Due to experimental error, 13 out of the 80 sessions were omitted from analyses of occupancy data as detailed in electronic supplementary material, table S2. A visual inspection of data trends indicates that this loss did not have the potential to substantially change any of the conclusions.

#### (ii) Phase 2

A repeated measures ANOVA was carried out on all choice proportion data from *phase 2,* as well as *t*-tests on pooled data from the two respective choice combinations over the 5 days.

## 3. Results

### (a) No evidence that goldfish prefer advance information in paradoxical choice

**Figure 2.**
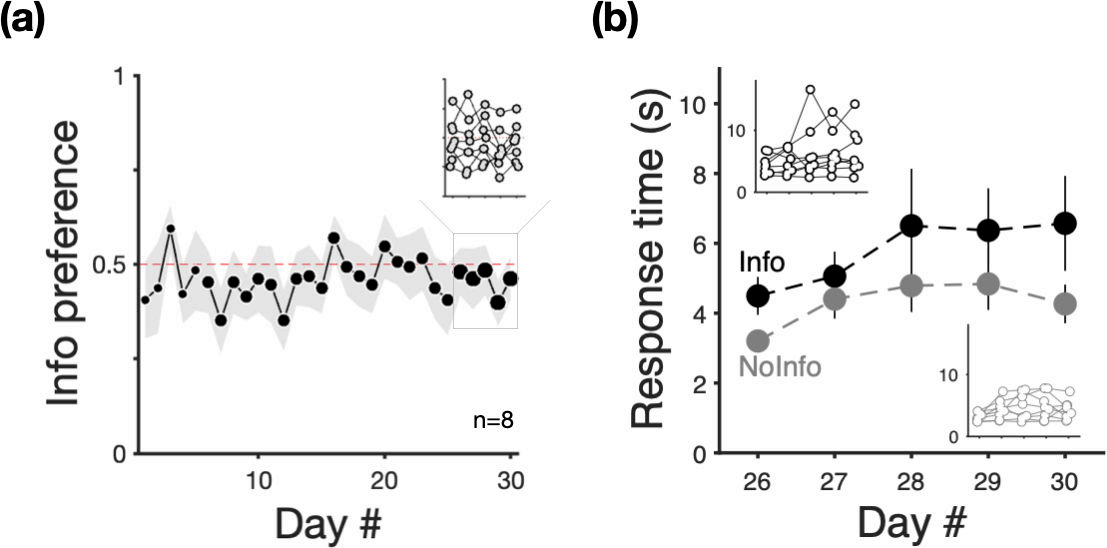
Goldfish preferences and response times for the informative alternative. (a) Mean proportion of choices for the informative option in two-option choice trials (*n* = 8; grey patch depicts ± s.e.m.). Marker size is proportional to the number of two-option choice trials; for details, see electronic supplementary material, table S1. (b) Mean response times (*n* = 8; in seconds ± s.e.m.) for responding to *Info* (black) and *NoInfo* (grey) from single-option trials that were intermixed with choice trials. In both panels, insets show individual animal means for the last 5 days of the experiment.

Our subjects showed no evidence of preference for the option in which outcomes were signalled between choice and trial end (figure 2a). Over 30 days, individual subjects were presented with over 500 choices: a one-way repeated measures ANOVA with day as a within-subject factor and proportion of choices for *Info* as the response variable showed no significant effect of day (F_29,203_ = 1.00, *p* = 0.471), indicating that, on average, the subjects did not develop a preference for either option. This lack of preference is also supported by Bayesian analyses of choice proportion data that find strong evidence against there being any differences between testing days (electronic supplementary material).

In addition to two-option choices, during this period, subjects were also presented with one-option forced trials. In these trials, we recorded the time latency from subjects starting a trial to proceeding to the single option presented to them; the ‘response time’. Response times are often a more sensitive metric of valuation than choice proportion in simultaneous trials, with shorter response times in one-option encounters associated with greater preference in choices [61–63]. We examined response times in forced trials with either alternative during the last 5 days of the experiment. Figure 2b shows that, on average, individuals took longer to respond in trials with the informative option than in trials with the non-informative option. This was reflected in a two-way repeated measures ANOVA with day and option as within-subject factors and response time as the response variable that showed a significant effect of day (F_4,28_ = 4.95, *p* < 0.01), and option (F_1,35_ = 9.09, *p* < 0.01), but no interaction (F_4,35_ = 0.217, *p* = 0.927). Consistent with previous studies (e.g. [63,64]), response times in forced trials and proportion of choices were related. Electronic supplementary material, figure S1 shows a negative correlation between individuals’ strength of preference for the informative option and the difference in response time between both options (individuals with shorter response times for a given option in forced trials show greater preference for that option in choices).

### (b) Post-choice goldfish occupancy shows discrimination of stimuli signalling future outcomes

**Figure 3.**
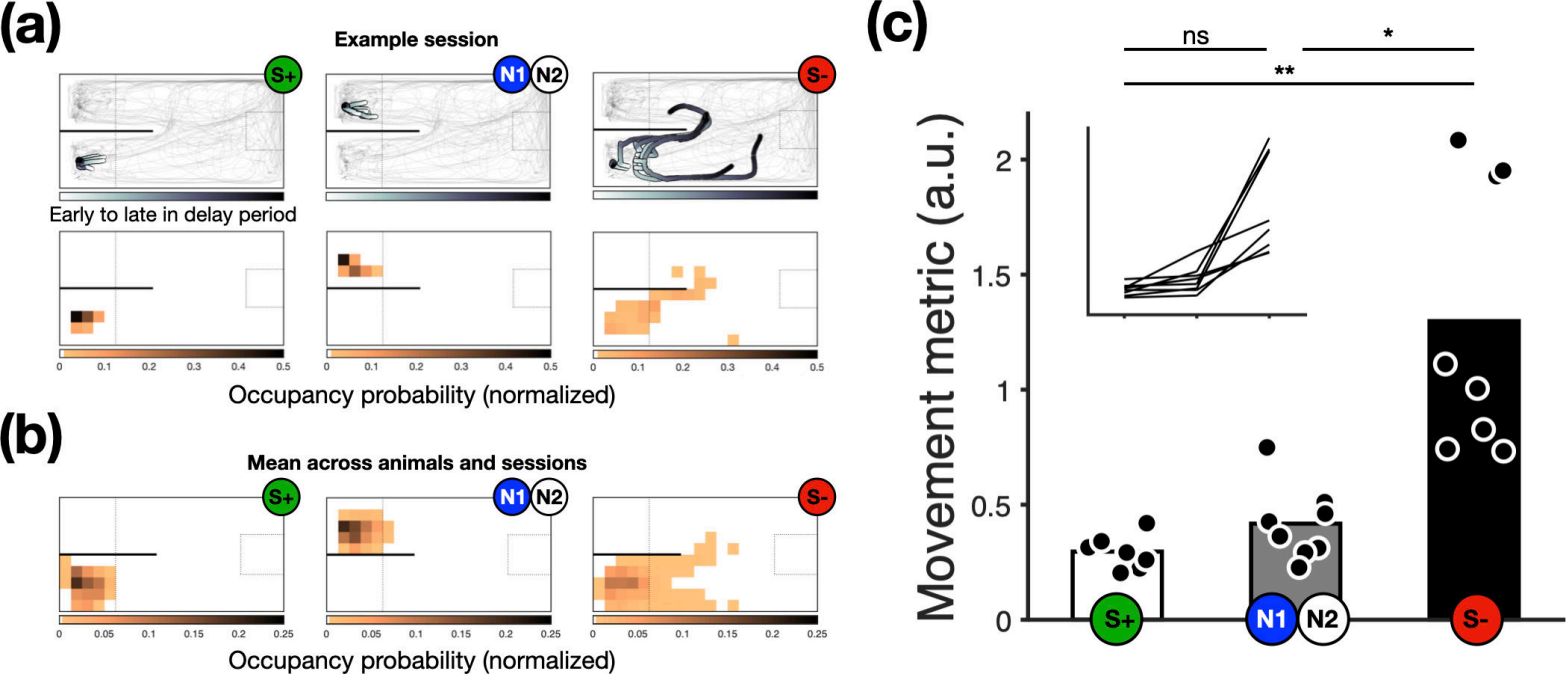
Goldfish spatial location varied according to cues’ reward contingencies. (a) Top row: trajectories of a representative animal and session during the post-choice delay period, split by trials in which the animal was presented with S+ (100% reward, left), N1 or N2 (50% reward, middle), or S− (0% reward, right). Darker trajectory shading shows the subject’s location further into the delay period. Thin grey lines show the trajectories for the entire session for reference. Middle row: corresponding normalized density heat maps of fish occupancy, darker shading indicates greater occupancy. (b) Same as above but averaged across all animals and days (*n* = 8 animals, 5 days each). For illustrative purposes, responses to *Info* stimuli appear on the left and *NoInfo* stimuli on the right, though this was counterbalanced across subjects. (c) Movement metric computed as the entropy of subjects’ spatial distributions (see *Methods* for details), where larger values correspond to more movement. Markers show individual animal means and bars the group averages. Inset shows individual trends connecting individual data points shown in the main figure (same scale). *p < 0.05, **p < 0.01.

To investigate whether the lack of preference between the options shown in choice trials was due to a putative inability of goldfish to learn the contingencies associated with either option, we examined subjects’ behaviour towards the outcome-predictive stimuli in each alternative. We focused on goldfish locomotion for the last 5 days, during a 4 s portion (cropping off 0.5 s after choice and 0.5 s pre-outcome) of the delay period preceding reward outcomes. This shows that goldfish discriminated between cues: proximity to the sites of reward delivery varied according to each cue’s reward contingency (recall that reward probabilities were S+100%; N1/N 50%; S− 0%). When reward was forthcoming (figure 3a,b left panels) or potentially forthcoming (figure 3a,b centre panels), subjects generally remained in the choice zone, close to the pellet dispenser. When the reward was not forthcoming (figure 3a,b right panels), subjects actively swam away from the choice zone and pellet dispenser, a trend shown for each subject (electronic supplementary material, figure S2).

To quantify the behavioural responses of subjects to post-choice reward-predictive stimuli, we calculated the entropy of subjects’ spatial probability distributions during the same post-choice delay (i.e. cropping off 0.5 s after choice and 0.5 s pre-outcome). Higher entropy values indicate subjects wandered over a larger portion of the experimental tank during this period. Data pooled from the last 5 days show that fish spatial occupancy during cue presentation corresponded to the probability of food delivery (figure 3c), indicating cue discrimination. The entropy of spatial distributions (mean ± s.e.m.) was lowest when S+ was presented (0.30 ± 0.024), higher when N1/N2 was presented (0.42 ± 0.058) and greatest when S− was presented (1.30 ± 0.21), reflecting that subjects spent more time near the site of food delivery when reward probability was greater. A one-way repeated-measures ANOVA on these data with stimulus as a within-subject factor and entropy as the response variable revealed a significant effect of stimulus (S+, N1/N2, S− ; F_2,14_ = 18.48, *p* < 0.001). *Post hoc* pairwise comparisons showed that the entropy of spatial distributions was significantly different between S+ and S− (*p* < 0.01), S− and N1/N2 (*p* < 0.05), but not between S+ and N1/N2 (*p* = 0.165), though subjects did show greater adherence to the site of reward delivery when S+ was present compared with N1 or N2. Overall, these results indicate that subjects did discriminate the contingencies and potentially developed an aversive response to S−. Interestingly, previous work in pigeons has emphasized the appetitive effect of S+ over the aversive effect of S− [37,38].

### (c) Goldfish choices between reward-predictive stimuli suggest biased weighting of reward probability

The results from *phase 1* show that subjects were indifferent between a site where their choice led to either S+ or S− and a site where the post-choice stimuli (N1 and N2) did not predict the trials’ outcome, but their post-choice behaviour showed discrimination between the stimuli themselves. This is evidence for learning the contingencies, but does not prove that goldfish can express such learning in their choice of feeding site. To corroborate whether they could express stimulus valuation as site preferences, in *phase 2* trials started directly with a presentation of pairs of stimuli that in *phase 1* had been experienced after their choices (electronic supplementary material, figure S3a).

When choosing between S+ and either N1 or N2, subjects were, on average, indifferent, while when choosing between S− and either N1 or N2, they had a clear preference for the latter. A two-way repeated measures ANOVA, with stimulus and day as within-subject factors and proportion of choices for S+/S− as the response variable, revealed a significant effect of stimulus (S+ or S−; F_1,35_ = 85.19, *p* < 0.0001), but no significant effect of day (F_4,28_ = 0.87, *p* = 0.0494), and no significant interaction (F_4,35_ = 1.13, *p* = 0.357; electronic supplementary material, figure S3b). Specifically, subjects showed no preference for S+ against N1/N2 (S+ choices: 46%, ± 0.072 s.e.m.; t7 = −0.63, *p* = 0.551), but preferred N1/N2 against S− (S− choices: 17%, ± 0.029; t7 = −8.97, *p* < 0.0001). Taken together, these results indicate that subjects could express their knowledge about S− by choosing away from it. This is consistent with results from *phase 1*, where there were similar locomotory responses to S+ and N1/N2, compared with S−, from which subjects moved away.

## 4. Discussion

**Figure 4.**
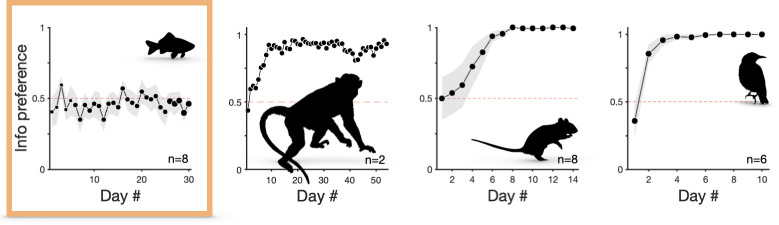
Preference for the informative alternative across four different species in similar paradoxical choice protocols. From left to right: goldfish (*Carassius auratus*; *n* = 8) from the present study (same as figure 2a). Rhesus macaque (*Macaca mulatta; n* = 2). Adapted with permission from [15, figure 1b]. Rats (*Rattus norvegicus*, *n* = 8). Adapted with permission from [6, figure 3]. European starlings (*Sturnus vulgaris*, *n* = 6). Adapted with permission from [24, figure 2]. Markers show group means and shading the s.e.m. The orange box highlights that the data are from the present study. Animal silhouettes from https://www.phylopic.org/ under Creative Commons licences.

In contrast to most vertebrate species previously tested (humans, monkeys, rats, pigeons and starlings), here, goldfish did not display a preference for signals foretelling probabilistic reward outcomes in the paradoxical choice task (figure 4), though they did discriminate between their contingencies.

Key to the interpretation of our results is the ability of subjects to discriminate between the outcome-predictive cues that distinguish the informative and uninformative alternatives. Two independent pieces of evidence suggest that the goldfish in our study did discriminate between the outcome-predictive stimuli. First, during the delay period between choice and outcome, when cues were present in the main experiment, subjects’ proximity to the site of reward delivery showed that they treated the cues according to their reward contingencies: the greater the likelihood of reward, the more time spent close to the site of food delivery (figure 3).

Similar discrimination has been shown in rats, but the latter did show a preference for the option offering advance information [6,19]. Second, in *phase 2*, we presented choices between signals that predicted the trial’s outcome (S+ or S−) and signals that did not (N1 or N2). In *phase 1,* these cues had only been presented after the choice of option, while in *phase 2,* fish could choose directly between them. The purpose of these tests was to establish whether choice indifference in the main experiment could be due to a lack of post-choice signal discrimination or an inability to express valuation as choice, as opposed to a lack of preference. In this phase, the fish were indifferent between signals for sure or a 50% chance of reward, but preferred signals for a 50% reward over a signal for reward absence. Interestingly, this result differs partly from results in starlings, which showed both a preference for sure over 50% chance reward and a preference for 50% over sure omission [24]. Indifference between 100 and 50% reward signals was unexpected but could be due to a ceiling effect: if valuation is a strongly concave function of reward probability, the differential attractiveness of medium and high probability contingencies may be undetectable. Our result is reminiscent of the observation reported by Stagner & Zentall [65], who found that pigeons preferred partial over continuous reinforcement and interpreted it as a suboptimal extreme form of risk propensity. This remains an interesting research topic in its own right.

As far as possible, our protocol retained critical features of the task used in other species. Furthermore, unlike studies where subjects incur reward losses by preferring the informative option [17,20,24,65–68], to avoid the possibility of masking preference for information due to sensitivity to loss, in our study, the informative and uninformative options were equally profitable.

From a procedural perspective, task parameters other than profitability (i.e. reward/delay [69]) can influence the strength of preferences in paradoxical choice tasks (for reviews, see [37,38]). These include the salience and modality of outcome-predictive stimuli [6,24,42,70], the length of delay between choice and reward outcomes (longer delays lead to greater *Info* preference: [13,18,66,71]), the proportion of forced trials (more forced trials increase preference: [22]) and the action subjects must make to express preference [72,73]. Therefore, it is possible that our results depend on specific experimental parameters. Particular experimental limitations are that the length of delay between choice and reward outcomes may have been too short to elicit a preference for the informative option, and the sample size may be too low for trends in preference to be detected. However, *a priori* it is not obvious why such details would have masked an underlying preference for information. In the first place, similar parameter sets and sample sizes have generated robust preferences in other species (e.g. [6,15,24]; figure 4 shows sample sizes of these studies). We designed our experiment so as to minimize the potential influence of specific parameter values, by using counterbalanced stimuli to which goldfish are known to respond [47] and selecting a delay duration (5 s) long enough to elicit preferences in monkeys (i.e. >2.5 s; [15,16,30,34]) but shorter than the 10 s used in rat, starling and pigeon studies, to reduce the possibility of task disengagement in the freely swimming fish. Furthermore, our subjects underwent training with one-option forced trials before two-option choice trials were introduced, and forced trials were interspersed throughout the main experiment, reducing the possible influence of side biases. These considerations, taken together with the fact that subjects learned to discriminate the cues associated with each option under our set of experimental parameters, lead us to believe that our results probably do reflect genuine species differences between goldfish and other vertebrate models so far tested.

Such species differences in preferences for informative stimuli offer insight into the mechanistic and functional underpinnings of paradoxical preferences so far observed in some bird and mammal species. Currently, a point of contention concerns the proximate motivation of observed preferences. One interpretation is that preferences reflect a form of information-seeking reminiscent of curiosity, driven by a reinforcing effect of uncertainty reduction. Another view is that preferences are a by-product of basic conditioning mechanisms that confer stimuli associated with positive outcomes’ secondary reinforcing properties, which do not mandate representations of uncertainty. We reasoned that if previously observed preferences are primarily driven by the latter—basic mechanisms of reinforcement learning shared across vertebrates—then we would also expect species showing associative learning capabilities (but not necessarily sensitivity to outcome uncertainty) to also display preferences for the informative alternative. Our finding that goldfish do not show preferences for the informative alternative, despite showing secondary conditioning in other contexts [50], indicates that conditioned reinforcement is not sufficient to promote preferences for *Info* in goldfish, and therefore raises the possibility that information-seeking may be underpinned by derived cognitive mechanisms in other species that do show *Info* preference. The conditioned reinforcement account posits that while positive cues become reinforcing, cues associated with negative outcomes do not acquire equally effective inhibitory properties for the informative alternative, meaning that overall, subjects would form stronger associations between choice and the informative option, but evidence from the present study is inconsistent with these assumptions.

For example, rat preferences for the informative option have been shown to be less robust than ‘other species’ [18,19,74], and it has been suggested that this is because rats experience conditioned inhibition from signals anticipating undesirable outcomes [74–76]. Some of our results indicate that this may also be the case in goldfish. In *phase 1*, when presented with S−, subjects swam away from the stimulus (figure 3; see also [77]), and in *phase 2,* subjects rarely selected S− when it was presented alongside other stimuli (electronic supplementary material, figure S3), suggesting that stimuli for reward omission can acquire aversive properties that could inhibit *Info* preference. Furthermore, in *phase 2*, subjects were indifferent between a stimulus for 100% reward versus one for 50% reward, suggesting that stimuli for sure reward may not acquire strong secondary reinforcing properties as suggested by the conditioned reinforcement account (but see [78]). Manipulations involving the removal of S− (e.g. [6,79,80]) could help to elucidate the extent to which conditioned inhibition plays a role in goldfish preferences for informative alternatives.

A potential explanation for a lack of preference for advance information in goldfish is that they lack sensitivity to reductions in uncertainty present in the mammal and bird species so far tested. This is speculative at this stage, but if confirmed, it would raise another theoretical challenge for future research: increased cognitive or neural sophistication, allowing handling of uncertainty, may also cause loss of foraging performance; species sophisticated enough to find information rewarding *per se* may choose suboptimally when allowed to seek for unusable information. This raises the question of how individuals trade off foraging for consumable resources with the general acquisition of environmental information.

Functionally, the lack of preference for advance information in goldfish may reflect a difference in foraging mode compared with other species so far tested. Vasconcelos *et al*. [24] argued that preferences for a lower pay-off alternative providing advance non-instrumental information in the lab may reflect adaptive mechanisms that are rate maximizing in natural circumstances, where information about forthcoming rewards will likely be usable. They envisaged that decision-makers could use such information to avoid unnecessary opportunity costs incurred while pursuing prey. The relevance of this line of reasoning to particular species, including carp that are primarily suction feeders and scavengers, can only be established through field research in ecological circumstances, but it is enough to sustain the view that paradoxical behaviour in the lab does not justify the epithet of ‘suboptimal’.

Another functional consideration is that a tendency to seek non-instrumental information is presumably linked with fitness costs in the form of lost foraging opportunities, predator exposure and energy expenditure. It has been argued that non-instrumental information-seeking behaviour allows individuals to acquire information that is not presently useful for a specific task, but may contribute to novel social and ecological problem-solving in the future. However, if there are not strong selection pressures for highly flexible, prospective behaviour, as may be the case in the ecological context of goldfish, fitness costs may preclude the emergence of robust information-seeking mechanisms. Further, goldfish (like experimental rats and mice, but not starlings) are laboratory bred and reared, and this may have modified their behaviour with respect to wild ancestors growing in ecological circumstances.

Our use of goldfish among the tens of thousands of diverse teleost fish was somewhat opportunistic, and given the breadth of this taxon, it would not be justified to claim that the results are generalizable to other, not yet investigated fish species. This constraint, of course, applies to the handful of mammals and birds studied so far, and one can only hope that further studies will bring more taxonomic definition. If, however, further research proves that secondary conditioning is present across multiple fish species that do not show preference for advance informative signals, while a variety of birds and mammals continue to confirm the current picture, questions about phylogenetic emergence would acquire relevance and importance: are there substantial and consistent differences in post-reptilian lifestyles that make information-seeking qualitatively more influential than it was before the split of tetrapods from the ancestral vertebrates trunk? If so, what are these differences? If nothing else, these ‘known unknowns’ add weight to the view that cognitive research benefits from escaping the use of a narrow set of laboratory species to potentiate a comparative, adaptive perspective.

## Data Availability

The code and all data that supports the findings of this study are available on Dryad [82]. Supplementary material is available online [83].
